# Vitamin E Dietary Supplementation Improves Neurological Symptoms and Decreases c-Abl/p73 Activation in Niemann-Pick C Mice

**DOI:** 10.3390/nu6083000

**Published:** 2014-07-30

**Authors:** Tamara Marín, Pablo Contreras, Juan Francisco Castro, David Chamorro, Elisa Balboa, Mònica Bosch-Morató, Francisco J. Muñoz, Alejandra R. Alvarez, Silvana Zanlungo

**Affiliations:** 1 Departamento de Gastroenterología, Facultad de Medicina, Pontificia Universidad Católica de Chile, Santiago 8330024, Chile; E-Mails: tamara.marin.m@gmail.com (T.M.); jcastros@uc.cl (J.F.C.); eibalboa@uc.cl (E.B.); 2 Departamento de Biología Celular y Molecular, Facultad de Ciencias Biológicas, Pontificia Universidad Católica de Chile, Santiago 8330025, Chile; E-Mails: prcontre@uc.cl (P.C.); ddchamor@uc.cl (D.C.); 3 Laboratory of Molecular Physiology and Channelopaties, Department of Experimental and Health Sciences, Universitat Pompeu Fabra, Barcelona 08003, Spain; E-Mails: monica.bosch@upf.edu (M.B.-M.); paco.munoz@upf.edu (F.J.M.); 4 Center FONDAP for Genome Regulation, Santiago 8370415, Chile

**Keywords:** vitamin E, Niemann-Pick C, cholesterol, lysosomes, apoptosis, c-Abl

## Abstract

Niemann-Pick C (NPC) disease is a fatal neurodegenerative disorder characterized by the accumulation of free cholesterol in lysosomes. We have previously reported that oxidative stress is the main upstream stimulus activating the proapoptotic c-Abl/p73 pathway in NPC neurons. We have also observed accumulation of vitamin E in NPC lysosomes, which could lead to a potential decrease of its bioavailability. Our aim was to determine if dietary vitamin E supplementation could improve NPC disease in mice. NPC mice received an alpha-tocopherol (α-TOH) supplemented diet and neurological symptoms, survival, Purkinje cell loss, α-TOH and nitrotyrosine levels, astrogliosis, and the c-Abl/p73 pathway functions were evaluated. In addition, the effect of α-TOH on the c-Abl/p73 pathway was evaluated in an *in vitro* NPC neuron model. The α-TOH rich diet delayed loss of weight, improved coordination and locomotor function and increased the survival of NPC mice. We found increased Purkinje neurons and α-TOH levels and reduced astrogliosis, nitrotyrosine and phosphorylated p73 in cerebellum. A decrease of c-Abl/p73 activation was also observed in the *in vitro* NPC neurons treated with α-TOH. In conclusion, our results show that vitamin E can delay neurodegeneration in NPC mice and suggest that its supplementation in the diet could be useful for the treatment of NPC patients.

## 1. Introduction

Niemann-Pick type C (NPC) is an inherited progressive neurovisceral disease [1,2]. This genetic disease is caused by the mutation of one of the genes encoding for the NPC1 or NPC2 proteins [2,3,4,5], which are involved in the transport of free cholesterol from the endosomal/lysosomal compartment to the rest of the cell. Although the detailed mechanism for cholesterol egress mediated by the NPC1 and NPC2 proteins is unknown, the currently accepted model is that NPC2 binds free cholesterol, during or after lysosomal hydrolysis of LDL cholesteryl esters, and subsequently, NPC1 mediates the exit of cholesterol from lysosomes [6,7]. Mutations in the *Npc1* gene (95% of NPC cases) and in the *Npc2* gene (5% of NPC cases) produce the same phenotype leading to accumulation of unesterified cholesterol and other lipids within lysosomes [2,8]. NPC patients present a broad range of clinical symptoms with variable age of onset and rate of progression including hepatosplenomegaly, vertical supranuclear gaze palsy, dysarthria, dystonia, cerebellar ataxia, and seizures [2]. One of the hallmarks of NPC disease is a progressive and extensive neurodegeneration which is caused by an increase in apoptosis [9]. Although there is a general loss of neurons in the Central Nervous System (CNS), cerebellar Purkinje cells are early and especially affected [1,10,11,12,13,14]. 

Recent evidences suggest that oxidative stress contributes to NPC cell death. Indeed, we and others have observed oxidative tissue damage in the liver and cerebellum of the *Npc1*^−/−^ mice together with a gene expression profile indicative of increased oxidative stress [15,16,17]. Moreover, NPC patients presented decreased antioxidant capacity and reduced Coenzyme Q10 in serum, which indicates a decrease in antioxidant defenses, along with elevated thiobarbituric acid-reactive species and carbonyl formation [18,19,20]. In addition, an increase in cholesterol oxidation products, such as 7-ketocholesterol (7-KC) and 3β,5α,6β-cholestanetriol (3β,5α,6β-triol) has been detected in NPC1 patients as well as in *Npc1*^−/−^ mice, whose levels seem to correlate with the severity and progression of the disease [21,22,23]. Interestingly, previous work from our group demonstrates that oxidative stress is the main upstream stimulus activating apoptosis in NPC neurons through the c-Abl/p73 proapoptotic pathway [24]. For instance, we have shown that treatment with the antioxidant *N*-Acetyl Cysteine (NAC) prevented c-Abl/p73 activation and apoptosis in *in vitro* NPC-like neurons [24]. However, oral supplementation of NAC only partially improved liver function and moderately reduced neurologic symptoms of *Npc1*^−/−^ mice [15,23]. Moreover, no significant effects on oxidative stress were detected, other than moderate improvement of the fraction of reduced CoQ10, in a short-term NAC therapeutic trial in NPC1 patients. Together, these results suggest a limited efficacy of NAC on NPC disease.

However, the beneficial effect of other antioxidants as therapeutic agents for NPC disease cannot be ruled out. Therefore, we wanted to analyze the effects of a diet supplemented with vitamin E on neurological symptoms of NPC mice. Interestingly, data from the literature and results from our lab showed increased levels of vitamin E, and in particular α-TOH, its most important biological derivative, in cerebellum and liver lysosomes of NPC mice [25,26]. Interestingly, one of the most damaged regions in NPC disease, the cerebellum, contains high levels of vitamin E [27]. Furthermore, mutations in the gene coding for the α-TOH transporter protein (α-Ttp) result in a neurologic syndrome of spinocerebellar ataxia called Ataxia with Vitamin E Deficiency or AVED. This condition, also observed in the α-Ttp knock-out mouse model, *Ttpa*^−/−^, is characterized by progressive ataxia, sensory loss and severe damage of Purkinje cells [28,29,30], sharing some symptoms with NPC patients. Moreover, recently Ulatowski *et al*. [26] reported the effect of vitamin E deficiency on the cerebellum of the *Ttpa*^−/−^ mice. They observed increased cerebellar oxidative stress and Purkinje cellular atrophy and decreased dendritic branching of Purkinje neurons that correlated with behavioral deficits in motor coordination and cognitive functions. Together, this data suggest that vitamin E transport could be altered in NPC cells affecting Purkinje neurons more strongly. In this sense, it is important to mention that a previous *in vivo* study showed that treatment with vitamin E, administered orally via gavage once a week, exerts a small but significant beneficial effect on the locomotor performance in NPC mice [31].

In this work, we analyzed the effect of vitamin E dietary supplementation on the *Npc1*^−/−^ mice evaluating neurological symptoms, survival, Purkinje cell loss, α-TOH and nitrotyrosine levels, astrogliosis, and the c-Abl/p73 pathway. The α-TOH rich diet delayed loss of weight, improved coordination and locomotor function and increased the survival of NPC mice. Moreover, we found preserved Purkinje neurons related to increased α-TOH levels and reduced levels of astrogliosis, nitrotyrosine and activated and phosporylated p73 in cerebellum. A decrease of c-Abl/p73 activation was also observed in *in vitro* NPC neurons treated with α-TOH.

Together these results show that vitamin E can delay neurodegeneration in NPC mice and suggest that its supplementation in the diet could be useful for the treatment of NPC patients.

## 2. Experimental Section

### 2.1. Animals

BALB/c mice carrying a heterozygous mutation in the *NPC1* gene (NPC mice) were kindly donated by Dr. Peter Pentchev. Genotypes were identified using a PCR-based screening as described previously by Amigo *et al*. [32], Wild-type (WT) and NPC male mice received control (Prolab RMH3000 (5P00), Labdiet, St. Louis, MO, USA) and α-TOH supplemented (vit E) diets (Prolab 5P00 w/2000 IU/kg vitamin E (5AU8), Labdiet) from postnatal day (p) 28. Homozygous mutants and the wild type mice eat roughly the same amount of food until 7 weeks of age. At this age NPC mice are in a steady state with respect to food intake and weight gain but not later, as described by Xie *et al*. [33]. One group of animals; WT control *n* = 6, WT vit E *n* = 9, NPC control *n* = 8, and NPC vit E *n* = 8, was used for body weight daily measuring, during the full period of treatment, as well as locomotor tests made once a week. Another group of 4 and 5 control-and vit E-treated NPC mice respectively was used for survival analysis. For immunofluorescence and immunohistochemistry cerebella analysis one group of mice (*n* = 3) was treated with the diets for 3 weeks and then sacrificed. 

All protocols were approved and followed local guidance documents generated by the ad hoc committee of Chile (CONICYT) and were approved by the Bioethics Committee of the School of Medicine from Pontificia Universidad Católica de Chile (CEBA Protocol # 10-017). They were in agreement with the US Public Health Service Policy on Humane Care and Use of Laboratory Animals recommended by the Institute for Laboratory Animal Research in its Guide for Care and Use of Laboratory Animals.

### 2.2. Locomotor Function Tests

Locomotor coordination was evaluated weekly during treatment using two tests specially validated for NPC mice [34]. In the Hanging test, the mouse was placed to hang at the center of a horizontal bar (3 mm diameter; 35 mm long) with forepaws. The body position of the animal was observed for 30 s and scored as described in Voikar *et al*. [34]. In the beam test mice were placed at the end of a beam (100 cm long; 2.5 cm wide). Animals were trained to finish the task as quickly as possible. The number of falls during the test was counted.

### 2.3. Tissue Immunohistochemical and Immunofluorescense Procedures

Mice (7 weeks-old) were anesthetized with xylazine/ketamine (0.12 and 0.8 mg/10 g body weight, respectively) and perfused with 4% paraformaldehyde in PBS. Cerebellum was removed and postfixed overnight at 4 °C, followed by 30% sucrose in PBS at 4 °C overnight, then were cut in 40 μm sagittal sections with a cryostat (Leica CM1850) at −20 °C. Four to five slices by animal were stained by experiment. We examined at least 3 animals per condition.

For calbindin immunohistochemical analysis, anti-calbindin D-28K antibody (AB1778, 1:1000; Chemicon International, Temecula, CA, USA) was used with the avidin-biotin-horseradish peroxidase complex method (Vector Laboratories, Burlingame, CA, USA).

To assess the extent of the microglial activation the tissues were incubated with PBS twice for 10 min. After pre-incubation in glycine 0.15 M for 10 min sections were incubated with blocking solution (BSA 5% in PBS) for 1 h, and incubated overnight at 4 °C with an anti-rabbit primary antibody against the glial fibrillary acidic protein (GFAP) (1:500, Sigma Chemical Co., St. Louis, MO, USA). After washing with PBS, sections were incubated for 1 h, at room temperature, with a goat anti-rabbit secondary antibody coupled to Alexa 555 (1:400, Invitrogen Detection Technologies, Carlsbad, CA, USA). Then tissues were washed with PBS and covered with Dako Fluorescent Mounting Medium (Dako, Carpintería, CA, USA). Images were captured with an Olympus BX51 microscope (Olympus, Tokyo, Japan) and analyzed with the Image-Pro Express program (Media Cybernetics, Bethesda, MD, USA).

For nitrotyrosine immunohistochemical analysis, free-floating cerebellum sections were blocked for 2 h at room temperature in a solution of 0.1 M phosphate buffer (PB) with 0.3% Triton X-100 and 3% normal goat serum. Then, sections were incubated overnight at 4 °C with mouse anti-nitrotyrosine antibody (1:100, MAB5404, Millipore Corporation, Billerica, MA, USA). After three 10-min washes in PB, sections were incubated for 2 h, at room temperature, with a goat anti-mouse secondary antibody coupled to Alexa 488 (1:500, Sigma Chemical Co.). Afterwards, sections were incubated for 10 min with a solution of TOPRO-3 iodide (1:1000, Molecular Probes, Eugene, OR, USA) for nuclear staining. Finally, after two 10min washes in PB, sections were mounted onto slides with Mowiol mounting media. Confocal images were obtained using a Leica SP2 confocal microscope.

### 2.4. Cell Culture, α-TOH, Imatinib and U18666A (U18) Treatments

The HT22 neuronal hippocampal cell line was maintained in Dulbecco’s modified Eagle’s medium (DMEM) supplied with 10% Fetal bovine serum (FBS) [35]. The cells were pre-treated with Imatinib at 5 µM or with α-TOH at 50 µM by 1 h. α-TOH was dissolved in analytical ethanol (vehicle), the stock concentration was 12 mM. For each experiment we used a fresh α-TOH solution. Later, cells were treated with U18 (Enzo Life Sciences Inc. Farmingdale, NY, USA) at 0.5 µg/mL for 24 h.

### 2.5. Filipin Staining

HT22 cells were fixed in 4% paraformaldehyde/4% sucrose in PBS for 30 min. After, cells were washed with PBS and treated with 1.5 mg/mL glycine for 20 min. Finally cells were treated with 25 µg/mL Filipin (Sigma Chemicals Co.) for 30 min, washed with PBS and covered with Fluoromount-G (Southern Biotech, Birmingham, AL, USA). Images were captured with an Olympus BX51 microscope (Olympus) and analyzed with the Image-Pro Express program (Media Cybernetics, Bethesda, MD, USA).

### 2.6. Immunofluorescence on Coverslips

HT22 cells were placed on poly-lysine-coated coverslips (30,000 cells/cover). After 2 days in DMEM/FBS, cells were fixed in 4% paraformaldehyde/4% sucrose in PBS and permeabilized with 0.02% Triton X-100. Then, cells were blocked with 10% bovine serum albumin in PBS. Immunostaining was carried out using anti-p-c-Abl Tyr^412^ (1:500; C5240, Sigma Chemical Co., St. Louis, MO, USA), anti-p73 (1:300; 5B1288, Abcam Inc., Cambridge, MA, USA), anti-β-tubulin (1:250, sc-9104, Santa Cruz Biotechnology, Santa Cruz, CA, USA) and Phalloidin: TRITC (1:5000; PF7551, ECM Biosciences, Versailles, KY, USA). Anti-rabbit-AlexaFluor-488 (1:1000) and anti-mouse-Alexa Fluor-555 (1:1000) (both from Invitrogen Detection Technologies) were used as secondary antibodies. Fluorescent images were captured with a confocal Olympus microscope or with an Olympus BX51 microscope (Olympus) and analyzed with the Image-Pro Express program (Media Cybernetics). Three coverslips were stained for each experimental group. We examined at least 5 images per coverslip in at least 3 independent experiments. For pixel quantification the Multi Measure application (ImageJ) was used. For the quantification of the images of the cultured HT22 cells each cell was taken as an independent region of interest (ROI). Then each ROI was averaged and divided by the total number of quantified neurons.

### 2.7. Western Blot Analysis

Proteins were prepared as described previously [36]. Cerebellar protein samples (60 μg) or HT22 protein samples (60 µg) were resolved by SDS-PAGE. The immunoblot was carried out using anti-p73 (H-79; 1:1000) (Santa Cruz Biotech), anti-c-Abl (K-12; 1:1000) (Santa Cruz Biotech), anti-ε-COP (obtained from Dr. Monty Krieger, Massachusetts Institute of Technology, Cambridge, MA, USA) (1:5000)) and anti-tubulin (T5168; 1:5000) (Sigma) antibodies, and secondary antibodies conjugated with horseradish peroxidase (1:3000) (Upstate Biotechnology, Lake Placid, NY, USA).

### 2.8. HPLC-EC α-TOH Content Measurements

Samples of frozen tissue (brain and cerebellum) from control- and vit E-treated NPC mice (*n* = 2) were weighed and then mechanically homogenized, placed in 0.5 mL homogenization buffer (20 mM Tris pH 7.2; 2 mM MgCl_2_; 0.25 M sucrose; 1 mg/mL Leupeptin; 1 mM Pepstatin; 1 mM PMSF and 0.1% BHT). Tissues were mechanically homogenized using an Ultraturrax (Kinematica, Littau, Suiza). α-TOH content was determined by reverse phase HPLC-EC as described by Motchnik *et al*. [37]. Briefly, the sample was resuspended in ethanol and mixed briefly. Afterwards, hexane was added. The solution was mixed, centrifuged for 15 min at 1000× *g*, and the upper hexane layer was transferred to a glass tube; the hexane extraction procedure was repeated twice. Hexane extracts were pooled and dried at room temperature under a stream of nitrogen, and the resulting pellet was dissolved in methanol/ethanol (1:1, v/v). Samples were then separated in columns using 20 mM Lithium Perchlorate in ethanol/H2O (96:4, v/v) as mobile phase.

### 2.9. Statistical Analysis

Mean and standard error of the mean values with the corresponding number of experiments are indicated in the figure legends. Probability values of the data for Student *t*-tests and ANOVA tests with Bonferroni’s post-test were obtained using the GraphPad Prism 5 (Graph Pad Software, Inc., San Diego, CA, USA).

## 3. Results

### 3.1. Vitamin E Treatment Increases Survival and Improves Locomotor Function of NPC Mice

**Figure 1:**
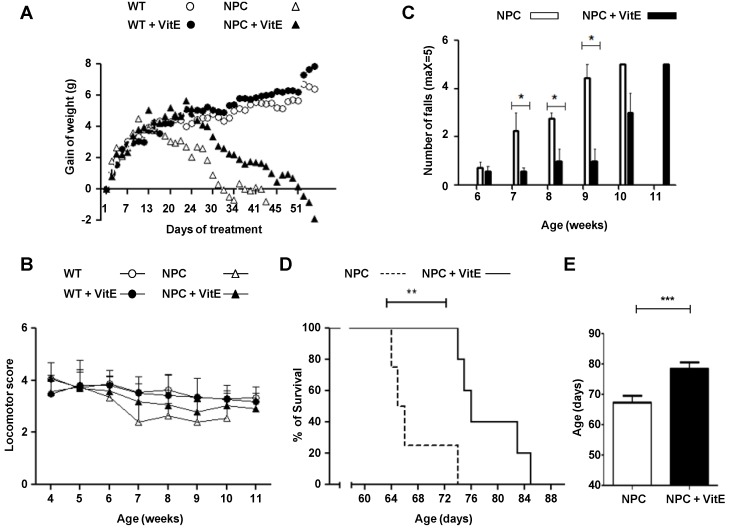
Vitamin E treatment increases survival and improves locomotor function of NPC mice. Wild-type (WT) and NPC mice received vitamin E (Vit E) (2000 IU/kg dl-alpha tocopherol acetate) or control diets starting at p28. (**A**) Weight was registered during the full period of treatment; (**B** and **C**) Motor coordination was assessed weekly by the hanging test and the beam test, respectively. For **A** and **B** white and black circles are shown for control- and Vit E-treated WT mice and white and black triangles are shown for control- and Vit E-treated NPC mice. For C white and black bars are shown for control- and Vit E-treated NPC mice. Control- and Vit E-treated WT mice did not fall down in this test (bars for these groups are not shown); (**D**) Survival was determined by continuing with the treatment until death. The dotted and solid lines are shown for control- and Vit E-treated NPC mice; (**E**) Mean survival age graphic. White and black bars are shown for control- and Vit E-treated NPC mice. Data are shown as mean ± SE, except for A in which SE are not shown to improve the visualization of each group. * *p* <0.05, ** *p* < 0.01, *** *p* < 0.0001. For **A**–**C** the following number of animals were used: WT control *n* = 6, WT Vit E *n* = 9, NPC control *n* = 8, and NPC Vit E *n* = 8. For D and E survival was measured in a group of 4 and 5 NPC mice fed with control and Vit E diets, respectively.

In order to test the effect of the vitamin E treatment, wild-type and NPC mice were fed with an α-TOH supplemented diet (2000 IU/Kg dl-Alpha Tocopherol Acetate) and a control diet starting at p28. Weight was registered during the full period of treatment (Figure 1A). The α-TOH rich diet delayed loss of weight in the NPC mice compared to those with the control diet. Weight started to decline at approximately 6 and 7 weeks of age in NPC mice fed control and α-TOH rich diets, respectively, and the rate of decline was reduced in the mice treated with the α-TOH rich diet (Figure 1A).

We evaluated coordination and locomotor function using two different tests (Figure 1B,C) at regular intervals throughout treatment. The α-TOH rich diet-treated NPC mice exhibited an improvement in the hanging test, which evaluates coordination of the four paws and the tail (Figure 1B). A delay in the onset of symptoms was observed in the α-TOH rich diet-treated NPC mice from 7 week of age.

In the beam test, which evaluates equilibrium and coordination, the α-TOH rich diet-treated NPC mice showed fewer falls than the control diet treated-NPC mice (Figure 1C). In this test, a significant improvement was observed from 7 weeks until 9 weeks of age. At 11 weeks of age, only NPC mice treated with the α-TOH rich diet survived.

Next, we analyzed the effect of α-TOH supplemented diet on the lifespan of NPC mice, starting the treatment from p28. Interestingly, α-TOH treatment significantly improved survival of NPC mice (Figure 1D). We observed a mean increase of 11 days in survival (Figure 1E). This increase in survival correlated with a tendency toward a greater corporal weight and improved locomotor function.

### 3.2. Vitamin E Treatment Improves Purkinje Cell Survival and Decreases Astrogliosis and Nitrotyrosine Levels in the Cerebellum of NPC Mice

**Figure 2:**
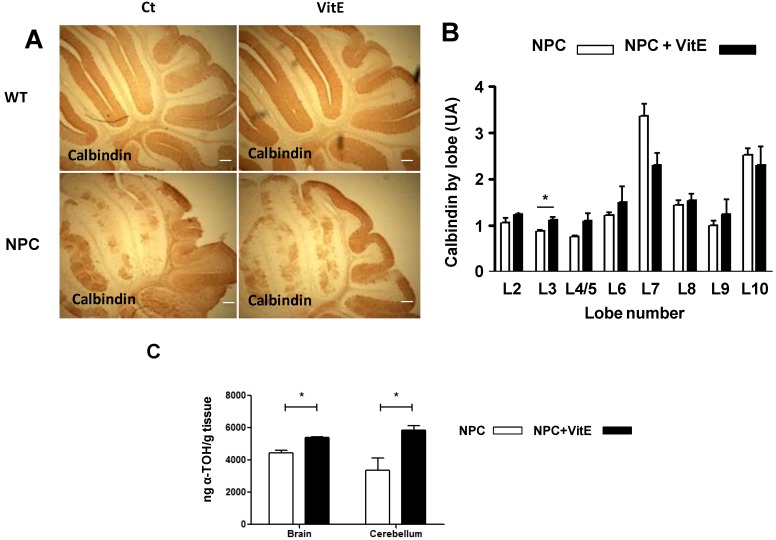
Vitamin E treatment improves Purkinje cell survival in the cerebellum of NPC mice. Wild-type (WT) and NPC mice received vitamin E (Vit E) (2000 IU/Kg DL-Alpha Tocopherol Acetate) or control (Ct) diets starting at p28. Cerebella from Ct- and Vit E-treated WT and NPC mice were analyzed at 7 weeks of age. (**A**) Calbindin was analyzed by immunohistochemistry. Scale bar, 100 μm; (**B**) A quantification of calbindin- immunoreactive Purkinje cell bodies in cerebellar sections (*n* = 3 mice/group) is shown; white and black bars are shown for Ct-and Vit E-treated NPC mice. Results are means ± SE. * *p* = 0.0091; (**C**) α-TOH content was measured by HPLC in brain and cerebellum. The following numbers of animals were used: Ct-and Vit E-treated NPC mice, *n* = 2 for brain and cerebellum analysis. Results are means ± SE. * *p* < 0.05.

Since vitamin E treatment improved neurological function in the NPC mice, we examined the effect of the α-TOH therapy on the survival of cerebellar Purkinje cells in mice treated for three weeks, from p28 to 7 weeks of age. Cerebella from control- and α-TOH-treated WT and NPC mice were analyzed for calbindin, a specific staining of cerebellum Purkinje cells, by immunohistochemistry. α-TOH treatment substantially improved Purkinje cell survival in NPC mice (Figure 2A). A quantification of calbindin-immunoreactive Purkinje cell bodies in cerebellar sections (*n* = 3 mice/group) is shown (Figure 2B). The greatest loss of Purkinje cells occurs in cerebellar frontal lobes, especially 2 and 4 [14]. Vitamin E treatment significantly improved Purkinje cell survival in lobe 3. Furthermore, the treatment had a trend to improve survival in lobe 2 and 4–5. In accordance with previous studies [38], lobes 7–10 from cerebella of NPC mice exhibited a marked resistance to cell death at 7 weeks of age, which was the time when the cerebella were analyzed.

In order to correlate the improvement in Purkinje cell survival with the vitamin E diet we measured α-TOH content in brain and cerebellum of control- and vit E-treated NPC mice. We found a significant increase in α-TOH levels in brain and cerebellum of vit E-treated NPC mice compared with control-treated NPC mice (Figure 2C).

**Figure 3:**
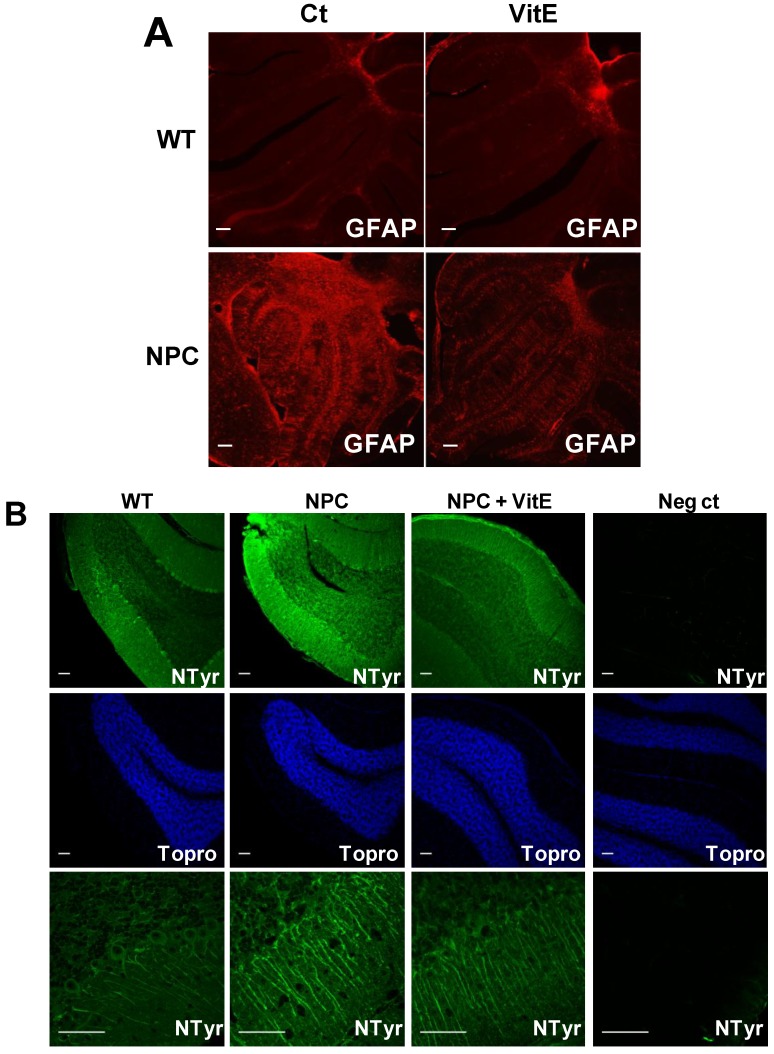
Vitamin E treatment decreases astrogliosis and nitrotyrosine levels in the cerebellum of NPC mice. Wild-type (WT) and NPC mice received vitamin E (Vit E) (2000 IU/kg dl-alpha tocopherol acetate) or control (Ct) diets starting at p28. Cerebella from Ct- and Vit E-treated WT and NPC mice were analyzed at 7 weeks of age; (**A**) Astrogliosis was analyzed by GFAP immunofluorescence. Scale bar, 100 μm. (**B**) Nitrotyrosine levels were determined using an anti-nitrotyrosine antibody and immunofluorescence analysis. A negative control (Neg ct) without the primary antibody was included. TOPRO-3 iodide was used for nuclear staining. Scale bar, 50 μm. 3 mice of each group (WT and NPC mice treated with Ct and Vit E diets) were used in each analysis.

We also analyzed astrogliosis and the oxidative stress marker nitrotyrosine using anti-GFAP and anti-nitrotyrosine antibodies, respectively, in the cerebellum of control-and α-TOH-treated WT and NPC mice. We found decreased levels of both markers in the cerebellum of the α-TOH-treated NPC mice (Figure 3A,B).

In summary, α-TOH-treated NPC mice showed an increase in Purkinje cell survival that correlated with increased α-TOH levels and decreased levels of GFAP and nitrotyrosine in the cerebellum.

### 3.3. Vitamin E Treatment Prevents U18666A Induced-cAbl/p73 and Caspase 3 Activation in HT22 Neurons and p73 Activation in the Cerebellum of NPC Mice

**Figure 4:**
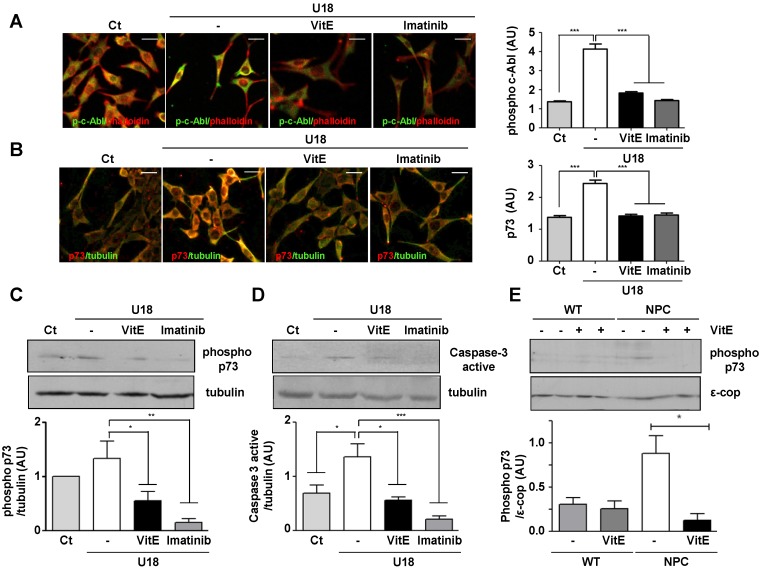
Vitamin E prevents U18666A induced cAbl/p73 and caspase 3 activation in HT22 neurons and p73 activation in the cerebellum of NPC mice. (**A**,**B**) Control (Ct) and U18-treated (0.5 μg/mL for 24 h) HT22 cells in absence or presence of vitamin E (Vit E) (50 μM) or Imatinib (5 μM) were fixed and immunostained using anti-p-cAbl, anti-p73, Phalloidin:TRITC and anti-β-tubulin antibodies. Data shown are an average of three independent experiments, and graphs shown quantifications, in arbitrary units (AU) of both phosphorylated (phospho)-c-Abl and p73 levels; (**C**,**D**) Immunoblot analysis of phosphoporylated (phospho)-p73 and caspase 3 active. Lysates from control and U18-treated (0.5 μg/mL for 24 h) HT22 cells in presence of vitamin E (Vit E) (50 μM) or Imatinib (5 μM) were resolved by 10% SDS-PAGE and analyzed by Western blot with anti-p-p73 and anti-caspase 3 active antibodies. Graphs show quantifications of phosphorylated (phospho)-p73 and caspase 3 active levels normalized by β-tubulin expression (arbitrary units, AU); (**E**) Immunoblot analysis of phospho p73 in NPC cerebellum. Cerebella homogenates from control- and Vit E-treated WT and NPC mice were analyzed at 7 weeks of age. The graph shows the quantification of p73 normalized by ε-cop expression (arbitrary units, AU). * *p* < 0.05, ** *p* < 0.01, *** *p* < 0.0001. Representative images of some experimental conditions are shown.

To complement our studies we evaluated the connection between vitamin E, the c-Abl/p73 proapoptotic pathway and NPC neurodegeneration, studying the expression of the phosphorylated and activated form of c-Abl and p73 in a pharmacological *in vitro* neuronal NPC model. We treated the HT22 neuronal hippocampal cell line with U18666A (U18) (0.5 μg/mL for 24 h), a well-known NPC phenotype inducer [39,40,41] that triggers significant cholesterol accumulation, in the presence of vitamin E (50 µM). In U18 treated cells we found dramatically increased p-c-Abl and p73 immunostaining relative to untreated cells. Interestingly, in the presence of vitamin E, p-c-Abl and p73 immunostaining were significantly decreased (Figure 4A,B).

On the other hand, we studied the levels of phosphorylated p73 and activated caspase 3 by Western blot analysis. We found that U18 increased both phosphorylated p73 and activated caspase 3 protein levels relative to untreated cells. Interestingly, we observed that the cells treated with U18 and vitamin E presented lower increases in phosphorylated p73 and activated caspase 3 expression (Figure 4C,D).

As a positive control for the inhibition of c-Abl and its signaling pathways, we used U18-treated HT22 cells in presence of Imatinib in both Western blot and immunofluorescence analysis [14]. As expected, we observed that Imatinib treatment significantly decreased p-c-Abl, p73, phosphorylated p73 and activated caspase 3 levels.

To evaluate the *in vivo* relevance of these findings we studied the expression of the phosphorylated and activated form of p73 in cerebellum homogenates of control- and α-TOH-treated WT and NPC mice by Western blot analysis (Figure 4E). We found that under the control diet, NPC cerebella had an approximately 2.5-fold increase with respect to wild-type cerebella. Moreover, we found a 4-fold decrease in the phosphorylated p73 protein levels in α-TOH treated NPC mice compared to the control diet treated-NPC mice.

In summary, these results show that vitamin E treatment prevented the activation of the c-Abl/p73 signaling pathway in NPC mice cerebellum and neurons.

## 4. Discussion

The most relevant finding of our study is that vitamin E treatment increased the quality of life of NPC mice by delaying body weight loss and neurological symptoms, decreasing cerebellar oxidative stress and astrogliosis and increasing Purkinje cell survival. Furthermore, vitamin E was supplemented on diet in relatively old p28 NPC mice and is a non-invasive therapy without apparent side effects. 

Interestingly, previous work from our group demonstrates that oxidative stress is the main upstream stimulus activating apoptosis in NPC neurons through the c-Abl/p73 proapoptotic pathway [24]. For instance, we have shown that treatment with the antioxidant *N*-Acetyl Cysteine (NAC) prevented c-Abl/p73 activation and apoptosis in *in vitro* NPC-like neurons [24]. However, oral supplementation of NAC only partially improved liver function and moderately reduced neurologic symptoms of *Npc1*^−/−^ mice [15,23]. These results suggested a limited efficacy of acute NAC intervention in NPC disease. Here we found that vitamin E can delay neurodegeneration in NPC mice and prevent c-Abl/p73 activation *in vivo* and in a neuron NPC model. However, treatment with vitamin E did not show positive effects on NPC mice liver (data not shown), whereas NAC treatment improved liver inflammation. These results support the idea that one of the major beneficial effects of the α-TOH diet is decreasing oxidative stress in the CNS. Accordingly, the levels of the oxidative stress marker, nitrotyrosine, were decreased in NPC mice cerebella with the α-TOH treatment.

Previous studies suggested that vitamin E transport could be altered in NPC cells, primary in Purkinje neurons. In fact, α-TTP (α-TOH transport protein) is expressed in Purkinje cells of patients having vitamin E deficiency or oxidative stress-associated diseases [42]. Moreover, we have previously published that NPC cells cannot transport vitamin E correctly leading to α-TOH buildup in the endosomal/lysosomal system. This may result in a decreased bioavailability and impaired antioxidant function of vitamin E in NPC, contributing to the disease pathogenesis [25,26]. Indeed, recently it was demonstrated that vitamin E is essential for Purkinje neuron integrity [43]. In this sense, a previous *in vivo* study showed that treatment with vitamin E, administered orally via gavage once a week, significantly delayed weight loss only in females. It also produced a significant but relatively small delay in the rate of disease progression in *Npc1*^−/−^ mice [31]. However, we show a more robust result with dietary vitamin E supplementation and using only male mice. Furthermore we observed an 11-day increase in NPC mice survival. Differences between this previous study and ours may be caused by the route and/or periodicity of vitamin E administration and by the gender of animal used. Although we obtained similar results in survival using the c-Abl inhibitor Imatinib [14], it is notable that in this work vitamin E supplementation was initiated relatively late, at p28, whereas improvement in NPC mice survival with Imatinib was achieved if treatment was initiated earlier, at p7 [14].

Our results demonstrated that a vitamin E supplemented diet caused a beneficial effect on Purkinje neurons and cerebellum, but we cannot rule out the possibility that the diet is improving other regions of the brain. The lobes in which we observed improvement are those that exhibit more cell death in NPC mice at the age in which the samples were taken [38].

Although vitamin E appears to have a protective function in the brain, relatively little is known about its mechanism of action or how it reaches the brain. Interestingly, the beneficial effects observed in NPC mice where achieved although vitamin E blood-brain barrier (BBB) penetrance is low in mice. Previous reports, in mouse brain showed small increases in α-TOH levels in response to a high dosage α-TOH supplementation when compared to those of plasma and liver. On average, the brain showed a 1.6-fold increase to 2 μM, as compared to the 6-fold increase in plasma to 4.9 μM and the 4.9-fold increase in liver to 240 μM [44]. This result suggests the presence of brain-specific mechanisms that limit α-TOH uptake into the brain, potentially at the BBB and apparently, the physiological benefit of supplementation was achieved despite the modest increase in cerebellar α-TOH [44].

On the other hand, in another mouse model, a very small increase in cerebellar α-TOH was physiologically relevant [43]. Accordingly, in this study authors showed that the average neuronal body size of the vitamin E-depleted *Ttpa*^−/−^ animals was markedly reduced by 25% compared to the *Ttpa*^+/+^ mice and long-term supplementation with vitamin E completely prevented this atrophy. This finding indicates that vitamin E deficiency contributes to marked atrophy of the Purkinje neurons, and that this fate was prevented by a high-dose, long-term supplementation with α-TOH. Apparently, the physiological benefit of supplementation was achieved despite the modest increase in cerebella α-TOH levels [43]. In addition, our results showed that after three weeks of dietary supplementation brain and cerebellum α-TOH levels were increased in NPC mice. Together our results and the data from the literature suggest that vitamin E is crossing the BBB and a modest increase in α-TOH levels is physiologically relevant.

Another possible scenario is that alterations in the BBB in the NPC mice, as well as in the *Ttpa*^−/−^ mice, which has been shown to improve with dietary vitamin E supplementation, allow its increased brain penetrance.

In this study we used a diet with high α-TOH content, and extrapolation to human treatment is difficult to determine. Further experiments are needed to determinate the effect and dose of vitamin E in NPC patients, because in humans serum α-TOH concentrations are poorly correlated with dietary vitamin E estimates and appear to be regulated by the α-TTP [45]. Moreover, determination of cerebellum physiological α-TOH relevant levels in NPC patients would be required.

As a major lipid-soluble antioxidant, α-TOH is essential for all cells due to its reactive oxygen species (ROS) scavenging activity [46]. However, vitamin E appears to be especially critical for CNS function. Little is known regarding the specific roles of α-TOH in the CNS, or the mechanisms by which it elicits its neuroprotective effects. Our results show a decrease in the levels of the well-accepted oxidative stress marker nitrotyrosine in α-TOH-treated NPC mice.

One possible target of α-TOH in NPC disease is mitochondria. Mitochondrial alterations have been reported in NPC cells [47,48] and in many neurodegenerative diseases [49,50] and studies have demonstrated that increased level of α-TOH in mitochondria is critical against oxidative stress in this organelle [51,52]. In fact, recent findings indicate that enrichment of mitochondria with protective α-TOH weakens production of ROS and antioxidant enzyme activities, especially in hepatic mitochondria [53]. This is important in NPC disease because the most affected organs are liver and brain. These organs are very active metabolically and contain the largest number of mitochondria. Furthermore another antioxidant that is decreased in NPC disease is GSH [54] and α-TOH prevented GSH depletion [55].

Another antioxidant function of α-TOH is counteracting the 7-ketocholesterol (7-KC) effect [56,57]. 7-KC is mainly produced from cholesterol autoxidation, and is increased in NPC disease [22]. On various cell types, 7-KC has often been shown to induce a complex mode of cell death by apoptosis associated with phospholipidosis. On murine oligodendrocytes treated with 7-KC the induction of apoptosis cell death was inhibited by α-TOH [56]. 

Interestingly, recent studies have shown that δ-tocopherol, a minor vitamin E specie, reduced lysosomal cholesterol accumulation, decreased lysosomal volume, increased cholesterol efflux, and alleviated pathological phenotypes in NPC1 fibroblasts [58]. These effects were correlated with a tocopherol-induced intracellular Ca^(2+)^ response and a subsequent enhancement of lysosomal exocytosis. 

Finally our results showing that vitamin E treatment, both *in vivo* and in neuronal NPC models, decreased the activation of the proapototic c-Abl/p73 pathway, also supports the idea that one of the major beneficial effects of the α-TOH diet is decreasing oxidative stress, but we cannot rule out other non-antioxidant actions of vitamin E.

## 5. Conclusions

In conclusion, our results show that vitamin E can delay neurodegeneration in NPC mice, and that this effect is related with its antioxidant capacity and suggest that its supplementation in the diet could be useful for the treatment of NPC patients.
